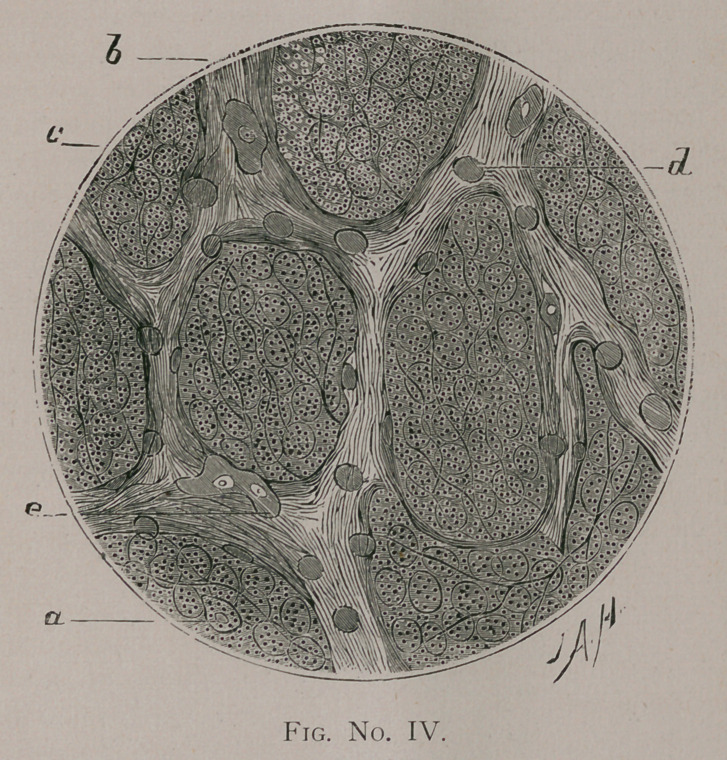# Pathological Changes in the Pleuro-Pneumonia or Lung Plague of Cattle

**Published:** 1880-01

**Authors:** W. H. Porter, J. Aycrigg Hegeman

**Affiliations:** Curator to the Presbyterian Hospital


### ART. III.—PATHOLOGICAL CHANGES IN THE PLEURO-PNEUMONIA OR LUNG PLAGUE OF CATTLE.

BY DR. W. H. PORTER,
CURATOR TO THE PRESBYTERIAN HOSPITAL,
AND
DR. J. AYCRIGG HEGEMAN.

OWING to the active measures which have recently been instituted by the State authorities, under the supervision of Gen. Patrick and Prof. James Law, of Cornell University, a large amount of pathological material and valuable data have been collected, which have enabled the authors to make a careful study of the gross and microscopic changes that take place in this insidious and most dangerous disease. Most of this material was in the possession of Dr. T. E. Satterthwaite, Lecturer on Comparative Pathology at the Columbia Veterinary College, and Dr. Allan S. Heath, Lecturer on the Diseases of Cattle at the same institution. It represented the findings in something over twenty necropsies of diseased cattle in New York City and adjacent parts. About the first naked-eye change that one sees is, either, on the one hand, a deep red mottling of the lung substance immediately beneath the pleura, or an infiltration in the interlobular spaces. It is well known that the pneumonic lobules in the cow are little separate blocks of tissue, that can be torn apart from one another, as the connective tissue holding them together is delicate and small in amount. In a lung that was examined very recently, the disease was evidently just beginning to attack a previously healthy lobe, and these two changes could be seen within a few inches of one another, while as yet the remainder of the lung was quite sound. Admitting this dual origin of the disease which such an example seems to establish, we may at once reconcile the opposing views of those pathologists who believe that the disease commences in the lungs, with the others who regard it as primarily a pleurisy. In the instance in question there was at one point a distinct effusion of fibro-purulent matter about and between a few lobules, while the lung substance appeared intact. In the other case the distinct mottling beneath the visceral pleura was found to be due to engorgement of the lobular vessels, with transudation of bright red coloring matter into the perivascular tissue. A little later, and the deep red color gave way to that of prune juice; but as yet there is no change in the inter-lobular tissue. This stage might be called that of vascular engorgement, and is probably at first confined only to the branches of the pulmonary artery in the lobule.

Later on, all the vessels, including the pulmonary veins and the capillaries, become dilated, engorged, and, as a result of this process, there is exudation of blood corpuscles and a new development of epithelium, and the lobule is swollen to four or five times its ordinary size, as we see it in health at post mortems. This is a stage that may be called the stage of infarction. Now there is an effusion of serum or lymph, perhaps even of sero-purulent fluid or pus, into the perilobular spaces. If the effusion is serous, it is apt to occur as a succession or chain of small cysts about the lobule, and this appears to be the usual change. There is an increase in the fibrillated connective tissue, which goes on increasing until, in some instances, it may surpass in thickness the lobule which is adjacent to it. In this way the lobules are compressed upon all sides by the gradual contraction of the connective tissue, and the result is inevitable death—necrosis—of the lung substance. Subjected to this enormous pressure, the alveolar structure is simply strangled. When this stage has been reached—the stage of necrosis—the lung tissue is generally thrown off *en masse*, a sharp line of demarcation separating it from the healthy tissue of the lung. It may be a whole lobe or only part of one. Usually the pleura, which has by this time taken an active participation in the process, forms a sac about the dead lung. This sac may contain clear or bloody serum, or a grumous offensive material. In advanced stages of the disease, as where the animal is in the stage of so-called convalescence, the necrosed lung may be compressed into a firm, yellow, round ball, the result of uniform pressure upon all sides by the fluid contained in the sac. It is not always that we find an exudation into the peri-lobular connective tissue. Sometimes the lobule may be in the condition of infarction, which is equivalent to red hepatization, and there may be no more than the ordinary connective tissue, generally more or less infiltrated with the liquid that has oozed from the lobule. On the other hand, we may find an extensive increase in the peripheral tissue, while the lobule preserves the ordinary pink color that belongs to healthy lung. In such cases we have usually an extension merely of the fibrinous exudation from a diseased part near by; or it may be that there was the usual congestion of the lobule, but the superabundant peri-lobular infiltration had so compressed the lobule that it could not reach the red hepatization stage of the usual type. Sometimes a peculiar condition is noticed in one lobe, as the anterior, where the posterior has been the seat of the disease. In this case the lobules are so compressed by the fluid in the sac, or the engorged lung, that they have been reduced to even a fourth of their normal size. The thickening of the pleura is a marked peculiarity, in many cases reaching as much as an inch in thickness. It often binds the lung down to the ribs, but sometimes does not. There is still another change which is constant in the necrotic stage—round about the air tubes, vessels and nerves is a tremendous exudation, which is four or five times that of the normal. Being fibrous, the tissue resists decay longest, and when the dead portion is torn open with the hand, a dendritic mass may be taken out, inprisoning in its rootlets larger or smaller portions of lung tissue. When washed, this substance plainly shows its arborescent character; the little rootlets terminate abruptly in little rounded knobs. These are the minute terminations of the bronchi, enclosed each by a little ball of fibrous tissue. At a far advanced part of the stage of necrosis, the lung tissue may have so far undergone dissolution, that it may have undergone a change into a pultaceous or, perhaps, a cheesy condition, while the firm inter-lobular network, and the peri or endo-bronchial formations either hang loosely in the fluid of the cavity, or have formed firm attachments to its walls. In rare cases the disease may be confined to a small number of lobules in the central portion of the lung. In such cases there may be no pleuritis, either visceral or parietal, but there will be pretty surely implication, more or less extensive, of the tissue about the diseased lobules—another instance of the propriety of our confining our nomen-

FIG. NO. I.

clature more closely to lung tissue proper. The organs of digestion are said to be dry. The third stomach is filled with dry food, as in other febrile diseases. Exudations of blood are said to take place into the large intestine. (Special Report No. 12, Department of Agriculture, 1879.) Miscroscopic examination sustains these statements. The methods employed were those which are commonly in use. The tissue was first soaked for forty-eight hours in Muller's Fluid; then in alcohol, until it was sufficiently hardened for cutting. Thus treated, some two hundred or more sections were made from different portions of the diseased lung, and for comparison, also a number from the healthy lung. The sections so prepared were carefully studied, and such as appeared to represent the essential lesions of the disease were selected. Accurate drawings were made of the various stages, including one of the normal lung. The drawings were made with unusual care by Dr. J. Aycrigg Hegeman,

FIG. NO. II.

the camera lucida being used in every instance. *Fig.* No. I. was drawn under a magnifying power of four hundred and fifty diameters; *Figs.* No. II. and III. of one hundred and sixty diameters; and *Fig.* No. IV. of one thousand diameters. Special attention was paid to the changes in the bronchus and its branches, and to determine whether the pneumonia was catarrhal or croupous. In the healthy lung the section of an air vescicle reveals little else than broad bands of fibrillated connective tissue (*Fig.* No. I. *c*), and the supporting net-work of elastic fibers (*Fig.* No. II.), with an occasional epithelial corpuscle on the alveolar wall. The corpuscles, however, do not show in either figure, but the normal lung structure is well shown, and will be of great service in comparison with the alveolar structure represented in *Fig.* IV. Taking for convenience, first the study of the changes around the bronchial tubes, or what may well be called a peri-bronchitis, one of the earliest changes that will be noticed, even before the pleura is much involved, is that the external or connective tissue coat of

FIG. NO. III.

the bronchus is thickened, and that there is a zone of inflammatory exudation around all the tubes, as in *Fig.* No. II. *b.*

The open alveolar space with normal lung structure is seen at *Fig.* II. *a*, where the alveolar tissue proper is not at all involved, and the lumen of the bronchus is perfectly free from exudation. With a higher magnifying power the epithelium is found normal and the cilia intact. Following the changes of the bronchus up to the point of the formation of abscess, this formation of new connective tissue will be enormous, varying from ten to twenty times more than normal, which condition is very perfectly, although only in part, represented in *Fig.* No. III.

At one portion of the figure an open space will be seen, which represents the lumen of the bronchus almost entirely free from debris of any kind. At *a* the cylindrical, ciliated epithelium is very distinctly seen; and at *b*, just behind the epithelial layer, is the abundant development of new formed connective tissue. When carefully examined with a high power this new connective tissue is found studded with enormously thickened capillary vessels. These vessels are often filled with blood corpuscles and little bodies, probably bacteria. There are also in this new connective tissue numerous paths or channels filled with small, round corpuscles about $\frac{1}{6,000}$ of an inch in diameter. The methods employed produced a shrinkage of fifty per cent. These channels at short intervals appear to be dilated; these dilitations being filled with the same small, round corpuscles represented in *Fig.* III. at *c*. The zone of small corpuscles, however, are not as definitely outlined as the figure represents. At some distance from the epithelial lining of the bronchus (*Fig.* III. *d*), the muciperous glands were seen. In one or two instances the duct leading into the bronchus was seen. In no case was any special change in the epithelium detected, the duct in every instance being open.

In the thickened pleura and interlobular tissue the same condition of newly formed connective tissue of blood vessels, and apparently of lymphatic channels with frequent dilatations, was observed. A direct communication between the lymphatics of the pleura and bronchus was not determined. In no case was the immediate alveolar wall, or any of the septa, found involved in this process; a careful search was made for evidence of croupous pneumonia in the alveoli, but in vain. The changes which were invariably seen are well represented in *Fig.* IV.

Nothing fibrinous was seen in the alveoli. Here the alveolar walls and septum are normal (*Fig.* IV. *b*), the alveoli are filled with swollen, granular, and in most instances indistinct epithelium (*Fig.* IV. *a* and *c*), but occasionally a very distinct epithelial corpuscle was observed, as will be seen at *d* and *e*. The blood corpuscles and fibrin are absent, and there is nothing indicative of croupous pneumonia. It is necessarily a catarrhal pneumonia. The healthy condition of the bronchial mucous membrane would lead one to suppose that the later changes in the air cells or alveoli were brought about largely by the rapid increase in the bronchus within, and the pleura without, thus compressing and cutting off the nutrition. It must be in this way that the larger abscesses are formed, leaving a main bronchus and its many

FIG. No. IV.

branches projecting into a softened mass of lung tissue or a cavity. It is difficult to decide whether in the early cases the mucous membrane is implicated in the very early manifestations of the disease. The evidence already collected goes to show that the poison is inhaled through the air passages. It is not improbable that the blood which is brought into such intimate connection with the air vesicles may be directly poisoned, and that, as a result, it may stagnate in the smaller vessels and capillaries, as was observed by the writers. It is well known that stasis is one of the prominent features in the blood of infectious disease. When once this condition is established, all the other symptoms that we have enumerated may follow one another in logical order.

The practical lessons of this study are plainly seen. The disease has no tendency to resolution with maintenance of the integrity of the lung. Its course is from bad to worse, and if both lungs are involved must clearly be fatal. When recovery takes place, as it doubtless does, it must be that the diseased portion has been confined in a cyst, or reduced to a caseous mass that is so firmly enveloped in its fibrous capsule as to be incapable of producing further harm. The writers have never seen any lung tissue that gave the promise of returning to its normal health condition. While, however, the disease exists, it is full of power for evil, and the State and National authorities should recognize the fact, and take prompt measures to root it out as other governments have done successfully, where they adopted thorough measures. Nothing will be so inexpensive in the end to the country as the destruction of every diseased animal, and with it, at the same time, every vestige of the diseased meat. Let no time be lost in enforcing these truths upon the community at large.

## Figures and Tables

**Fig. No. I. f1:**
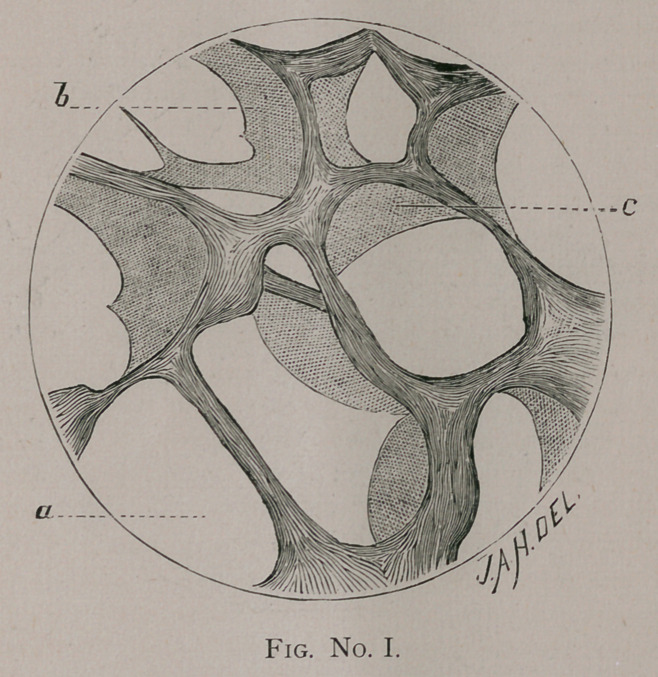


**Fig. No. II. f2:**
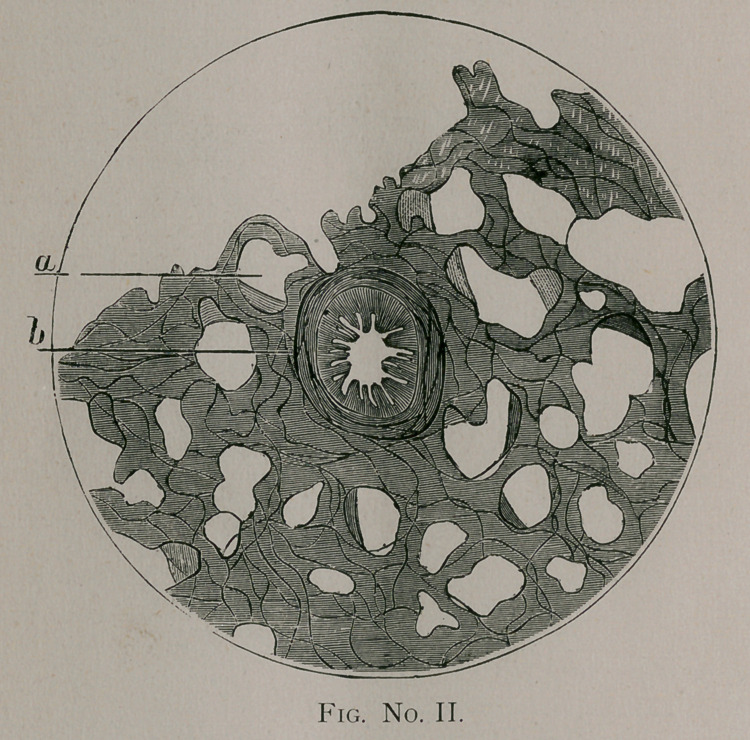


**Fig. No. III. f3:**
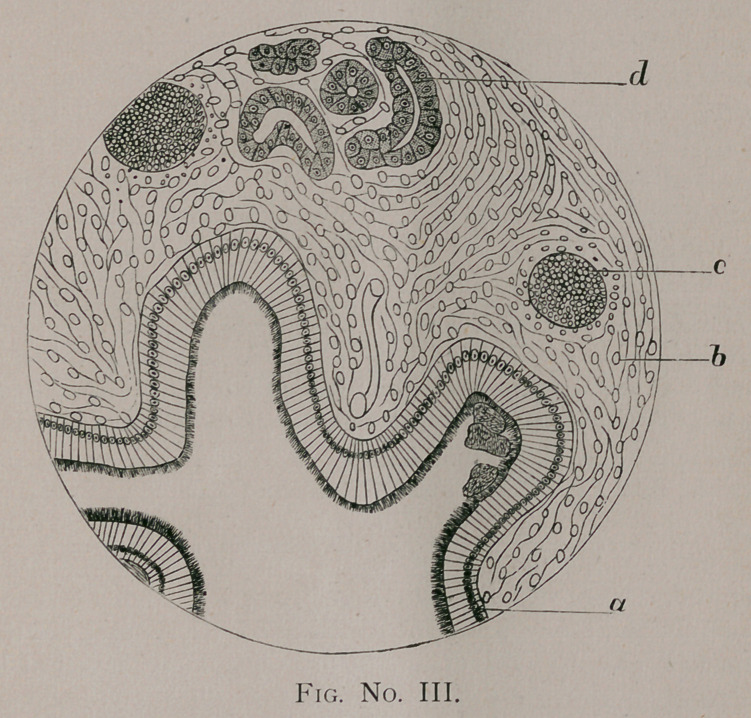


**Fig. No. IV. f4:**